# Suppressor of Cytokine Signalling 5 (SOCS5) Modulates Inflammatory Responses during Alphavirus Infection

**DOI:** 10.3390/v14112476

**Published:** 2022-11-09

**Authors:** Lukasz Kedzierski, Abigail Er Qi Tan, Isabelle Jia Hui Foo, Sandra E. Nicholson, John K. Fazakerley

**Affiliations:** 1 Department of Microbiology and Immunology, at the Peter Doherty Institute for Infection and Immunity, The University of Melbourne, Melbourne, VIC 3000, Australia; 2 Faculty of Veterinary and Agricultural Sciences, at the Peter Doherty Institute for Infection and Immunity, The University of Melbourne, Melbourne, VIC 3000, Australia; 3 Inflammation Division, The Walter and Eliza Hall Institute of Medical Research, Parkville, VIC 3052, Australia; 4 Department of Medical Biology, The University of Melbourne, Parkville, VIC 3010, Australia

**Keywords:** alphavirus, SOCS, encephalitis

## Abstract

CNS viral infections are one of the major causes of morbidity and mortality worldwide and a significant global public health concern. Uncontrolled inflammation and immune responses in the brain, despite their protective roles, can also be harmful. The suppressor of cytokine signalling (SOCS) proteins is one of the key mechanisms controlling inflammatory and immune responses across all tissues including the brain. SOCS5 is highly expressed in the brain but there is little understanding of its role in the CNS. Using a mouse model of encephalitis, we demonstrate that lack of SOCS5 results in changes in the pathogenesis and clinical outcome of a neurotropic virus infection. Relative to wild-type mice, SOCS5-deficient mice had greater weight loss, dysregulated cytokine production and increased neuroinflammatory infiltrates composed predominantly of CD11b^+^ cells. We conclude that in the brain, SOCS5 is a vital regulator of anti-viral immunity that mediates the critical balance between immunopathology and virus persistence.

## 1. Introduction

Central nervous system (CNS) virus infections are an important cause of morbidity and mortality worldwide and a significant global public health concern [1]. Arthropod-borne viruses (arboviruses) are major causes of encephalomyelitis in humans and include New World alphaviruses such as Western equine encephalitis (WEE), eastern equine encephalitis (EEE), and Venezuelan equine encephalitis (VEE) viruses [2]. Old World alphaviruses such as chikungunya (CHIKV), Ross River (RRV), or Mayaro (MAYV) viruses cause arthritogenic disease [3]. Therapeutic options to treat these diseases are limited and often ineffective, and as the spread of neurotropic viruses intensifies, there is a need to understand the mechanisms responsible for disease severity.

The brain is an immuno-specialised organ. Virus infection of the brain leads to activation of resident neural cell populations, such as microglia and astrocytes, recruitment of inflammatory cells and initiation of adaptive immune responses. These inflammatory responses, if left unchecked, can cause damage to brain tissue [4]. A key regulatory mechanism, which prevents these responses from becoming excessive is the suppressors of cytokine signalling (SOCS) [5]. The SOCS family is composed of eight members, SOCS1 to 7 and CISH (cytokine-inducible Src-homology 2 protein) [6]. SOCS proteins are negative regulators involved in the control of cytokine networks responsible for innate and adaptive immune responses [7]. In the CNS, SOCS proteins are expressed by resident cells and by infiltrating immune cells [8,9]. While the physiological role and mechanism of action of SOCS1 and SOCS3 are well understood, much less is known about the other family members (SOCS4-7). SOCS5 shares a high degree of amino acid homology with SOCS4 [10] and regulates epidermal growth factor receptor (EGFR) signalling [11]. We have previously demonstrated that SOCS5 plays a pivotal role as a regulator of inflammation in the lungs during influenza virus infection, restricting virus in the airways via regulation of EGFR and PI3K signalling [12]. Other studies have implicated SOCS5 in type I interferon (IFN) regulation during the infectious bursal disease virus [13] and feline herpesvirus [14] infections. The resting brain expresses high levels of SOCS5 [15,16], but there are limited data on its role during viral CNS infections. In vitro studies show that Japanese encephalitis virus (JEV) infection upregulates SOCS5 production in a human microglial cell line, but suppresses it in a mouse neuronal cell line, in each case leading to enhanced virus replication [17,18].

In the present study, we used SOCS5 knockout (*Socs5^−/−^*) mice to investigate the role of SOCS5 in virus encephalitis. We used the well-characterised Semliki Forest virus (SFV) mouse infection model [19] to demonstrate that SOC5 deficiency was associated with increased brain inflammation and exacerbated neuropathology. Our data show, for the first time, that SOCS5 is an important suppressor of immune and inflammatory responses that reduces immunopathology in the brain.

## 2. Materials and Methods

### 2.1. Animals

Generation of *Socs5^−/−^* mice on a C57BL/6 background [20] have been described previously. Animal experiments followed the NHMRC Code of Practice for the Care and Use of Animals for Scientific Purposes guidelines and were approved by the University of Melbourne Animal Ethics Committee (AEC 1714184). All mice were monitored daily for clinical signs including determination of body weight.

### 2.2. Virus

The avirulent A7(74) strain of Semliki Forest virus was used in this study [21]. All mice were inoculated intraperitoneally (i.p.) with 5 × 10^3^ pfu of virus in 0.1 mL PBS containing 0.75% bovine serum albumin (PBSA). TCID50 assays were used to titrate infectious viruses as described previously [22].

### 2.3. Tissue Sampling

To remove blood from the tissue vasculature following terminal anaesthesia, animals were perfused with PBS through the left cardiac ventricle. Brains were removed and processed for virus infectivity assay and chemokine/cytokine levels (half-brain bisected sagitally along the midline), for preparation of RNA for gene expression studies (half-brain), analysis of inflammatory cell infiltrates (entire brain), or for histopathological assessment.

### 2.4. Gene Expression Analysis

Half-brain specimens were submerged in RNA stabilisation reagent, RNAlater (Qiagen, Hilden, Germany). RNA was extracted using the RNeasy Lipid Tissue Mini kit (Qiagen, Hilden, Germany) according to manufacturer’s instructions and stored at −80 °C until use. RNA quantity was determined by spectrophotometry (NanoDrop 2000, Thermo Fisher Scientific, Waltham, MA, USA). 5 µg of RNA was converted to cDNA using SuperScript III reverse transcriptase (Invitrogen, Carlsbad, CA, USA). RT-PCR was carried out using Fast SYBR Green Mastermix (Thermo Scientific, Waltham, MA, USA) and primers recognising the NSP3 region of the virus genomic RNA: viral NSP3 reverse primer- 5′-GGGAAAAGATGAGCAAACCA-3′; viral NSP3 forward primer- 5′-GCAAGAGGCAAACGAACAGA-3′. Levels of virus RNA were normalised to the housekeeping gene, GAPDH. SOCS5 was amplified using previously published primer sequences [23]. Samples were run on a CFX96 real time PCR machine (Bio Rad, South Granville, NSW, Australia) with the following PCR conditions: the first cycle: 5 min at 95 °C, 10 sec at 95 °C, 15 sec at 60 °C and 1 sec at 72 °C. The following 45 cycles: 10 sec at 95 °C, 15 sec at 60 °C, 1 sec at 72 °C and ended with 10 min at 40 °C. The relative amount of virus genomic RNA was calculated using the 2^−∆∆CT^ method.

### 2.5. Immunophenotypic Staining

Single cell suspensions were purified from the brain and spleen. The brain tissue was digested with 1785 units/mL collagenase type III (Worthington, Lakewood, NJ, USA) and 6 units/mL DNase I. CNS-infiltrating leukocytes were isolated from the brain samples by centrifugation on a Percoll (Sigma-Aldrich, St. Louis, MO, USA) gradient (70%, 37%, and 30% Percoll). Purified cells were stained with the following antibodies: CD8-PerCP Cy5.5 (clone 53-6.7, BD Biosciences, San Diego, CA, USA), CD4-Pacific Blue (clone RM4-5, BD Biosciences), CD44-APC Cy7 (clone IM7, BD Biosciences), CD62L-PE Cy7 (clone MEL-14, BD Biosciences), CD103-APC (clone 2E7, Biolegend, San Diego, CA, USA), CD69-FITC (clone H12F3, BD Biosciences), PD-1-BV785 (clone 29F.1A12, Biolegend), KLRG-1-APC (clone 2F1, BD Biosciences), NK1.1-PE (clone PK136, BD Biosciences), CD3-FITC (clone 145-2C11, Biolegend), B220-APC Cy7 (clone RA3-6B2, BD Biosciences), IgD-PE Cy7 (clone 11-26c.2a, Biolegend), CD19-APC (clone 6D5, Biolegend), CD138-PE (clone 231-2, BD Biosciences), F4/80-FITC (clone BM8, Biolegend), CD11c-FITC (clone HL3 BD Biosciences), Gr1-FITC (clone RB6-8C5, BD Biosciences), Ly6C-PerCP Cy5.5 (clone AL-21, BD Biosciences), MHCII-Pacific Blue (clone AF6-120.1, Biolegend), CD45-APC Cy7 (clone 30-F11, BD Biosciences), Ly6G-PE Cy7 (clone 1A8, BD Biosciences), CD11b-APC (clone M1/70, Invitrogen), and CD11c-PE (clone HL3, BD Biosciences). Cells were stained for 30 min on ice, washed twice and analysed by flow cytometry on a BD Canto or BD Fortessa (BD Biosciences, San Diego, CA, USA), and analysed by FlowJo software (BD Biosciences, San Diego, CA, USA). Gating strategy is provided in Supplementary Materials (Supplementary Figure S2).

### 2.6. Cytokine and Chemokine Analysis

Cytokine and chemokine levels in the brain were analysed using the LEGENDPlex Multi-Analyte Flow Assay Kit (Biolegend, San Diego, CA, USA) Mouse Anti-Virus Response Panel (13-plex) according to manufacturers’ instructions.

### 2.7. Histopathology

Brains were removed from PBS perfused animals, fixed in 4% paraformaldehyde in PBS, processed for embedding in paraffin wax, cut into thin sections, stained with haematoxylin and eosin, and analysed microscopically. Histopathology was performed by the Phenomics Australia Histopathology and Digital Slide Service at the University of Melbourne. 

### 2.8. Statistical Analyses

Statistical analyses were performed using non-parametric Kolmogorov–Smirnov test or Student’s unpaired *t*-test within GraphPad Prism 9 software (San Diego, CA, USA).

## 3. Results and Discussion

SOCS5 has been shown to be an important regulator of antiviral immunity and inflammation in extra-neural tissues [12]. Therefore, we hypothesised that SOCS5 has an important role in controlling brain inflammatory and immune responses. To test our hypothesis, we used a Semliki Forest virus (SFV) mouse model system [19]. Following intraperitoneal infection and systemic replication, SFV infects the CNS, targeting predominantly neurons and oligodendrocytes [24]. The virus is effectively controlled by the humoral immune response, and the infectious virus is cleared from the brain by day 8 post-infection. However, brain virus RNA is detectable for many months and has the ability to give rise to infectious viruses in immunosuppressed animals [22]. SOCS5 is highly expressed in mouse brain in the cortex and cerebellum (Supplementary Figure S1), therefore, we investigated its expression kinetics in the brain of C57BL/6 mice following infection with SFV. Total RNA was purified at different times post-infection, transcript levels were normalised to a housekeeping gene GAPDH, and fold-changes were determined compared to uninfected controls. GAPDH has been chosen as a housekeeping gene based on previous reports [25,26], but has not been validated in the current study.

**Figure 1:**
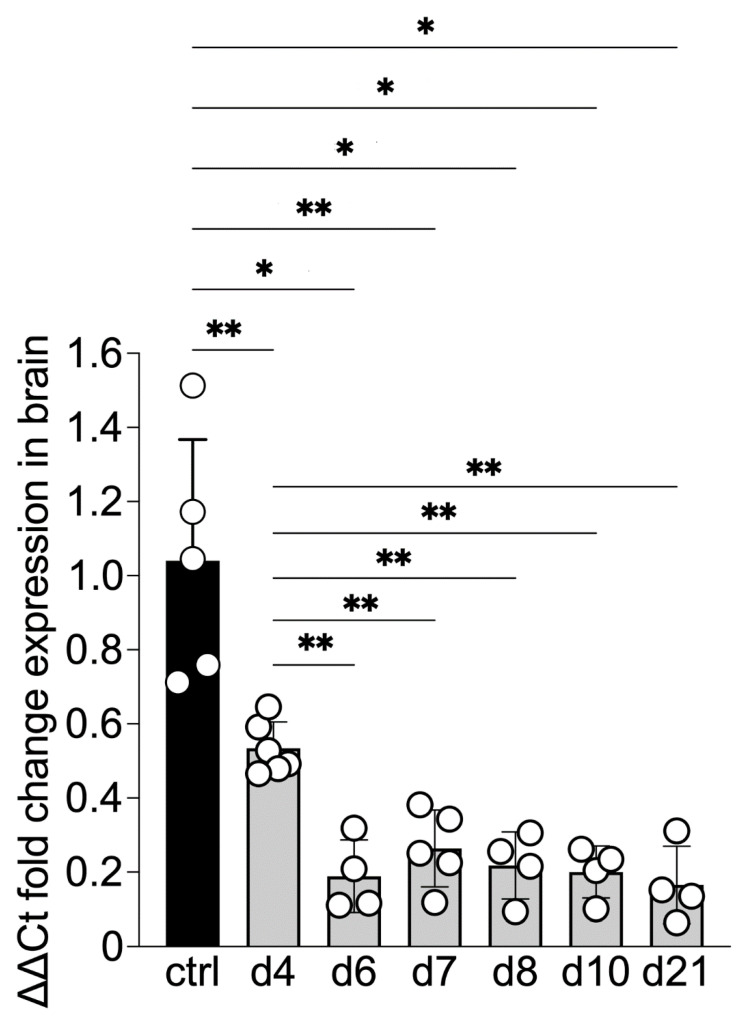
*Socs5* expression in the brains of C57BL/6 mice during the course of infection with SFV. Gene expression was normalised to GAPDH and the ΔΔCt method used to calculate log_10_ fold change relative to the untreated control. Each bar represents the mean of 4–6 biological replicates, error bars represent SD. Significance was determined by non-parametric Kolmogorov–Smirnov test, * *p* < 0.05, ** < 0.005.

SOCS5 transcripts were significantly downregulated by day 4 post-infection (dpi) and remained so until at least d21 (Figure 1). SOCS5 was also found to be suppressed in a mouse neuronal cell line upon infection with JEV [18]. This suggests that the virus may suppress SOCS5 expression as part of its strategy to avoid the host’s immune responses.

**Figure 2:**
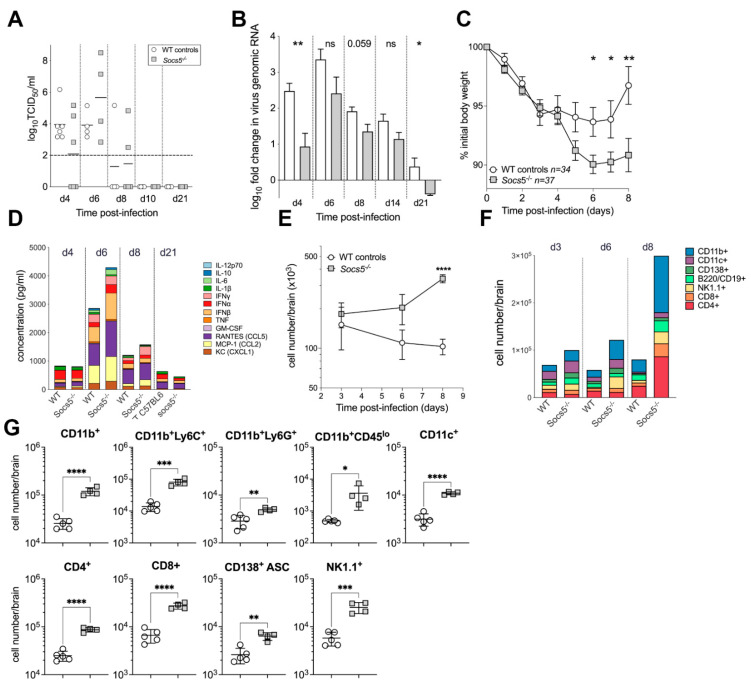
SOCS5 deficiency exacerbates clinical disease and increases brain inflammatory responses. (**A**) Titres of infectious virus in the brain during the course of SFV infection in *Socs5^−/−^* and wild-type C57BL/6 in mice. Viral titres were determined by TCID50 assay, the dashed line indicates the limit of detection. (**B**) SFV genomic RNA copies measured by qPCR in brain tissue during the course of infection with SFV in *Socs5^−/−^* and wild-type C57BL/6 mice. Transcript levels were normalised to a housekeeping gene GAPDH and fold-changes of genomic viral RNA were calculated compared to uninfected, naïve control. Each bar represents the mean of 4–6 biological replicates, error bars represent SEM, white bars—WT controls, grey bars—*Socs5^−/−^* mice. Significance was determined by unpaired Student’s *t*-test, * *p* < 0.05, ** < 0.005. (**C**) *Socs5^−/−^* mice showed significantly greater weight loss than C57BL/6 controls on 6–8 dpi. Combined data from 3 independent experiments are shown (*n* = 34 for C57BL/6, *n* = 37 for *Socs5^−/−^* mice). Error bars represent SEM, significance was determined by unpaired Student’s *t*-test, * *p* < 0.05, ** < 0.005. (**D**) Cytokine and chemokine protein levels were analysed by Legendplex in brain homogenates at different timepoints post-infection. Mean data are shown for biological replicates (*n* = 4–6). (**E**) Inflammatory cells in the brains of *Socs5^−/−^* and C57BL/6 mice during the course of infection with SFV. Data shown are from a time course experiment (biological replicates *n* = 4–7 per time point), error bars represent SEM, significance was determined by unpaired Student’s *t*-test, **** *p* < 0.0001. (**F**) Cellular composition of the inflammatory cells in the brains of *Socs5^−/−^* and C57BL/6 mice during the course of infection with SFV. Phenotype and cell numbers were determined by flow cytometry as described in Materials and Methods. (**G**) Significantly different brain cell populations on 8 dpi. Error bars represent SD, significance was determined by unpaired Student’s *t*-test, * *p* < 0.05, ** < 0.005, *** < 0.001, **** *p* < 0.0001.

To examine the in vivo role of SOCS5 during encephalitis, we intraperitoneally (i.p.) infected wild-type C57BL/6 and *Socs5^−/−^* mice with 5 × 10^3^ pfu of the A7(74) strain of SFV. Mice were monitored for clinical symptoms, and the infectious brain virus, viral RNA load, cytokine, chemokine, and inflammatory cell profiles were analysed. Relative to wild-type mice, levels of the infectious virus were lower in *Socs5^−/−^* mice at 4 dpi, with only three out of six mice having a detectable infectious virus in the brain (Figure 2A). This correlated with a significantly reduced viral RNA load in *Socs5^−/−^* mice at 4 dpi (Figure 2B). Thereafter, while there was a general trend towards lower levels of virus RNA in the brains of *Socs5^−/−^* mice, there was no significant difference in either the infectious virus or the virus RNA load (Figure 2B). Mice lacking SOCS5 lost significantly more body weight following SFV infection compared to wild-type control mice (Figure 2C). Severe weight loss is not generally associated with SFV infection, but there was considerable (>5%) and significantly greater weight loss in *Socs5^−/−^* mice than in wild-type mice at days 6, 7, and 8 post-infection. In the influenza mouse model, weight loss correlates with inflammatory responses at the site of infection [25]. Analysis of the cytokine and chemokine milieu of the brain (Figure 2D) demonstrated a similar profile of cytokines and chemokines at 4 dpi, at which time there was no difference in weight loss. On 6 dpi, the first day with a significant difference in weight loss, levels of cytokines and chemokines were higher than at 4 dpi, and higher in the *Socs5^−/−^* mice than in the wild-type mice. This difference was not statistically significant, but four cytokines were noticeably (approx. 2-fold) elevated in *Socs5^−/−^* mice compared to wild-type controls; IL-6 (185 vs. 82 pg/mL), RANTES (1248 vs. 762 pg/mL), IFNα (297 vs. 157 pg/mL), and IFNβ (920 vs. 518 pg/mL). These cytokines are all associated with early responses to viral infection.

Relative to wild-type mice, *Socs5^−/−^* mice had a significantly greater influx of immune cells into the brain on 8 dpi (Figure 2E). Phenotypic profiling of inflammatory cells (Figure 2F) showed that numbers of CD11b^+^ cells were significantly (*p* < 0.0001) higher in the *Socs5^−/−^* than in the wild-type mice. This included significantly higher numbers of neutrophils (CD45^hi^ CD11b^+^ Ly6G^+^*, p* = 0.04), inflammatory monocytes (CD45^hi^ CD11b^+^ Ly6C^+^*, p* = 0.03), and microglia (CD45^lo^ CD11b^+^ Ly6G^−^ Ly6C^−^, *p* = 0.02). There were also significantly higher numbers of antibody secreting cells (ASCs) (CD138^+^ B220^lo^ IgD^−^*, p* = 0.001), NK1.1^+^ cells (*p* = 0.0003), CD11c^+^ cells (*p* < 0.0001), and both CD4^+^ (*p* < 0.0001) and CD8^+^ (*p* < 0.0001) T cells (Figure 2G). 

**Figure 3:**
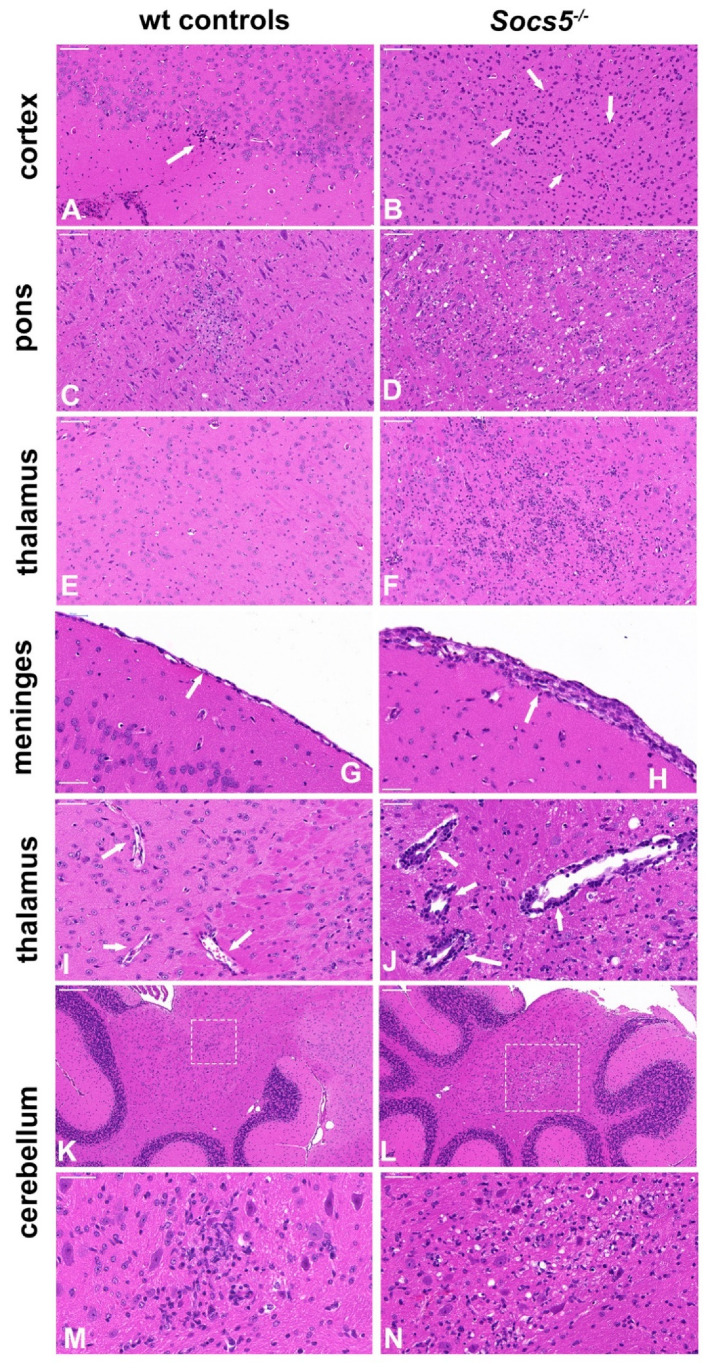
*Socs5^−/−^* mice show exacerbated brain pathology following SFV infection. Brains from *Socs5^−/−^* mice and control mice were removed (*n* = 2 for each genotype) on 8 dpi, sectioned sagittally and stained with HE to assess inflammation and neuronal degeneration; representative images are shown. Neuronal degeneration in the cortex (**A**,**B**), vacuolation in pons (**C**,**D**), gliosis in thalamus (**E**,**F**), meningeal lymphocytic infiltration (**G**,**H**), perivascular lymphocytic cuffing (**I**,**J**). Cerebellar vacuolation and infiltration (**K**–**N**), images (**M**,**N**) are magnified areas (outlined) of images (**J**,**K**), respectively. Bar = 100 µm (**A**–**D**); 50 µm (**E**–**J**,**M**,**N**); 200 µm (**K**,**L**). Arrows point to described histopathological changes (pyknotic nuclei in (**A**,**B**), cell infiltration in (**G**,**H**) and cuffing in (**I**,**J**)).

To evaluate the neuropathological changes in *Socs5^−/−^* (*n* = 2) and wild-type (*n* = 2) mice on 8 dpi, haematoxylin and eosin (HE) stained sections from the brains were assessed and scored. Overall, the meningoencephalitis was more severe in SOCS5-deficient mice than in C57BL/6 control mice (Figure 3). Changes consisting of multifocal lesions showing neuropil vacuolation, gliosis, necrosis of a few glial cells, moderate meningeal lymphocytic infiltration, and axonal swelling were observed in wild-type mice (Figure 3A–N). In contrast, brains of *Socs5^−/−^* mice displayed marked vacuolation of grey and white matter (status spongiosus) with axonal swelling, neuronal necrosis, more diffuse gliosis, perivascular lymphocytic cuffing, and substantial meningeal lymphocytic infiltration. There was also evidence of multifocal parenchymal necrosis with pyknosis, karyolysis, and karyorrhexis of neuronal and glial nuclei (Figure 3A–N).

The CNS can initiate and sustain strong inflammatory and immune responses upon viral infection [27]. Inflammation is a critical host defence mechanism, but it can also be damaging if not regulated. This is particularly the case in the brain which has minimal capacity for regeneration. A series of interlocked immune mechanisms controls infection and immunopathological damage [5]. Negative regulation of pro-inflammatory cytokine signalling is pivotal in these processes and the SOCS family proteins play a key role. Our data indicate that following a neurotropic alphavirus infection, SOCS5 is an important regulator of inflammatory responses.

Following SFV infection SOCS5 was downregulated in mouse brains infected with SFV and its levels remained low even after the infectious virus was cleared. This suggests that SOCS5 is a contributor to the immunosuppressive environment of the resting brain and is downregulated in response to viral infection. We have previously reported that SOCS5 is suppressed in primary human airway epithelial cells by the highly pathogenic avian H5N1 influenza virus [12]. Similarly, suppression of SOCS5 has been reported during JEV infection [18]. In the latter case, SOCS5 downregulation led to activation of its target, the EGFR [11,12,28], reduction of IFNβ production, and higher viral titres in the brain. In the present study, apart from an early reduction in brain virus titres, perhaps related to increased levels of type I interferons (IFNα and IFN β), there were no clear differences in SFV infectivity titres in the brain. Collectively, these data indicate that systemic SOCS5 deficiency (rather than in vivo inhibition as in [18]), had minimal effect on the dynamics of the SFV brain infection. However, systemic lack of SOCS5 led to increased chemokine and cytokine production in virus infected brains. This was mainly RANTES and IL-6 but also type-I interferons. SOCS5 suppresses EGFR phosphorylation. In the brain, activation of EGFR leads to activation of astrocytes and microglia [29,30], both of which produce cytokines/chemokines. Our results indicated overproduction of immunomodulators and exacerbated gliosis in *Socs5^−/−^* mice consistent with overactivation of these glial cell types, possibly caused by unregulated phosphorylation of EGFR due to the lack of SOCS5. In CNS viral infections, chemokines such as RANTES and cytokine such as IL-6 facilitate lymphocyte and monocyte recruitment. We observed significantly higher numbers of neutrophils, inflammatory monocytes, and microglia in *Socs5^−/−^* brains, which is consistent with the overall more severe meningoencephalitis changes. There were also significantly increased numbers of ASCs in the brains, but this had no apparent impact on infectious viral clearance. CD8^+^ T cell numbers were increased in *Socs5^−/−^* mice, and this could have been responsible for the reduction in the levels of virus RNA [22].

This study describes, for the first time, a phenotype for *Socs5^−/−^* mice in the context of a neuroinvasive alphavirus. While the absence of SOCS5 protein did not have a major impact on the kinetics of SFV infection in the brain, it resulted in higher levels of cytokines and chemokines in the brain and a greater loss of body weight. The higher levels of cytokines and chemokines were followed by increased numbers of inflammatory cells in the brain. Neuropathological changes in the brain were consistent with increased inflammatory infiltrates and cytokine production. These results indicate that SOCS5 plays a negative regulatory role in brain inflammatory and immune responses, although the exact mechanisms of its action in the CNS are currently unknown. It is obvious that SOCS proteins represent an important mechanism for maintaining homeostasis and controlling brain inflammation. Thus, our study adds key insights to the current knowledge regarding the physiological role of SOCS5.

## Supplementary Materials

The following supporting information can be downloaded at: https://www.mdpi.com/article/10.3390/v14112476/s1, Figure S1: SOCS5 localisation in the brain. Figure S2: Gating strategy for flow cytometric analysis of brain infiltrates. References [20,31] were cited in the supplementary materials.

## References

[1] Muñoz L.S., Garcia M.A., Gordon-Lipkin E., Parra B., Pardo C.A. (2018). Emerging Viral Infections and Their Impact on the Global Burden of Neurological Disease. Semin. Neurol..

[2] Barba M., Fairbanks E.L., Daly J.M. (2019). Equine viral encephalitis: Prevalence, impact, and management strategies. Vet. Med..

[3] Baxter V.K., Heise M.T. (2020). Immunopathogenesis of alphaviruses. Adv. Virus Res..

[4] Klein R.S., Hunter C.A. (2017). Protective and Pathological Immunity during Central Nervous System Infections. Immunity.

[5] Yang M.-S., Min K.-J., Joe E. (2007). Multiple mechanisms that prevent excessive brain inflammation. J. Neurosci. Res..

[6] Linossi E.M., Babon J.J., Hilton D.J., Nicholson S.E. (2013). Suppression of cytokine signaling: The SOCS perspective. Cytokine Growth Factor Rev..

[7] Alexander W.S. (2002). Suppressors of cytokine signalling (SOCS) in the immune system. Nat. Rev. Immunol..

[8] Baker B.J., Akhtar L.N., Benveniste E.N. (2009). SOCS1 and SOCS3 in the control of CNS immunity. Trends Immunol..

[9] Steffensen M.A., Fenger C., Christensen J.E., Jørgensen C.K., Bassi M.R., Christensen J., Finsen B., Thomsen A.R. (2014). Suppressors of Cytokine Signaling 1 and 3 Are Upregulated in Brain Resident Cells in Response to Virus-Induced Inflammation of the Central Nervous System via at Least Two Distinctive Pathways. J. Virol..

[10] Linossi E.M., Calleja D.J., Nicholson S.E. (2018). Understanding SOCS protein specificity. Growth Factors.

[11] Nicholson S.E., Metcalf D., Sprigg N.S., Columbus R., Walker F., Silva A., Cary D., Willson T.A., Zhang J.-G., Hilton D.J. (2005). Suppressor of cytokine signaling (SOCS)-5 is a potential negative regulator of epidermal growth factor signaling. Proc. Natl. Acad. Sci. USA.

[12] Kedzierski L., Tate M.D., Hsu A.C., Kolesnik T.B., Linossi E.M., Dagley L., Dong Z., Freeman S., Infusini G., Starkey M.R. (2017). Suppressor of cytokine signaling (SOCS)5 ameliorates influenza infection via inhibition of EGFR signaling. eLife.

[13] Fu M., Wang B., Chen X., He Z., Wang Y., Li X., Cao H., Zheng S.J. (2018). MicroRNA gga-miR-130b Suppresses Infectious Bursal Disease Virus Replication via Targeting of the Viral Genome and Cellular Suppressors of Cytokine Signaling 5. J. Virol..

[14] Zhang J., Li Z., Huang J., Yin H., Tian J., Qu L. (2019). miR-26a Inhibits Feline Herpesvirus 1 Replication by Targeting SOCS5 and Promoting Type I Interferon Signaling. Viruses.

[15] Wang J., Campbell I.L. (2002). Cytokine signaling in the brain: Putting a SOCS in it?. J. Neurosci. Res..

[16] Walker D.G., Whetzel A.M., Lue L.-F. (2015). Expression of suppressor of cytokine signaling genes in human elderly and Alzheimer’s disease brains and human microglia. Neuroscience.

[17] Sharma N., Kumawat K.L., Rastogi M., Basu A., Singh S.K. (2016). Japanese Encephalitis Virus exploits the microRNA-432 to regulate the expression of Suppressor of Cytokine Signaling (SOCS) 5. Sci. Rep..

[18] Hazra B., Kumawat K.L., Basu A. (2017). The host microRNA miR-301a blocks the IRF1-mediated neuronal innate immune response to Japanese encephalitis virus infection. Sci. Signal..

[19] Fazakerley J.K. (2004). Semliki Forest virus infection of laboratory mice: A model to study the pathogenesis of viral encephalitis. Emergence and Control of Zoonotic Viral Encephalitides.

[20] Brender C., Columbus R., Metcalf D., Handman E., Starr R., Huntington N., Tarlinton D., Ødum N., Nicholson S.E., Nicola N.A. (2004). SOCS5 Is Expressed in Primary B and T Lymphoid Cells but Is Dispensable for Lymphocyte Production and Function. Mol. Cell. Biol..

[21] Fazakerley J., Pathak S., Scallan M., Amor S., Dyson H. (1993). Replication of the A7(74) Strain of Semliki Forest Virus Is Restricted in Neurons. Virology.

[22] Fragkoudis R., Dixon-Ballany C.M., Zagrajek A.K., Kedzierski L., Fazakerley J.K. (2018). Following Acute Encephalitis, Semliki Forest Virus is Undetectable in the Brain by Infectivity Assays but Functional Virus RNA Capable of Generating Infectious Virus Persists for Life. Viruses.

[23] Kolesnik T.B., Nicholson S.E., Nicholson S.E., Nicola N.A. (2013). Analysis of Suppressor of Cytokine Signalling (SOCS) Gene Expression by Real-Time Quantitative PCR. JAK-STAT Signalling: Methods and Protocols.

[24] Fragkoudis R., Tamberg N., Siu R., Kiiver K., Kohl A., Merits A., Fazakerley J.K. (2009). Neurons and oligodendrocytes in the mouse brain differ in their ability to replicate Semliki Forest virus. J. NeuroVirol..

[25] Kedzierski L., Linossi E.M., Kolesnik T.B., Day E.B., Bird N.L., Kile B.T., Belz G.T., Metcalf D., Nicola N.A., Kedzierska K. (2014). Suppressor of Cytokine Signaling 4 (SOCS4) Protects against Severe Cytokine Storm and Enhances Viral Clearance during Influenza Infection. PLoS Pathog..

[26] van den Pol A.N., Robek M.D., Ghosh P.K., Ozduman K., Bandi P., Whim M.D., Wollmann G. (2007). Cytomegalovirus induces interferon-stimulated gene expression and is attenuated by interferon in the developing brain. J. Virol..

[27] Ahmed M.M., Johnson N.R., Boyd T.D., Coughlan C., Chial H.J., Potter H. (2021). Innate Immune System Activation and Neuroinflammation in Down Syndrome and Neurodegeneration: Therapeutic Targets or Partners?. Front. Aging Neurosci..

[28] Kario E., Marmor M.D., Adamsky K., Citri A., Amit I., Amariglio N., Rechavi G., Yarden Y. (2005). Suppressors of Cytokine Signaling 4 and 5 Regulate Epidermal Growth Factor Receptor Signaling. J. Biol. Chem..

[29] Liu B., Chen H., Johns T.G., Neufeld A.H. (2006). Epidermal Growth Factor Receptor Activation: An Upstream Signal for Transition of Quiescent Astrocytes into Reactive Astrocytes after Neural Injury. J. Neurosci..

[30] Qu W.-S., Liu J.-L., Li C.-Y., Li X., Xie M.-J., Wang W., Tian D.-S. (2015). Rapidly activated epidermal growth factor receptor mediates lipopolysaccharide-triggered migration of microglia. Neurochem. Int..

[31] Elefanty A.G., Begley C.G., Hartley L., Papaevangeliou B., Robb L. (1999). Scl Expression in the Mouse Embryo Detected with a Targeted Lacz Reporter Gene Demonstrates Its Localization to Hematopoietic, Vascular, and Neural Tissues. Blood.

