# Supplementary material for: Suppressor of Cytokine Signalling 5 (SOCS5) Modulates Inflammatory Responses during Alphavirus Infection
Source: Viruses. 2022 Nov 9;14(11):2476. doi: 10.3390/v14112476 (PMC9692489; doi:10.3390/v14112476)
Supplement: Supplementary file 1 [file viruses-14-02476-s001.zip › viruses-1978010 - supp conv.pdf]

viruses

Supplementary Materials

Suppressor of Cytokine Signalling 5 (SOCS5) Modulates Inflammatory Responses during Alphavirus Infection

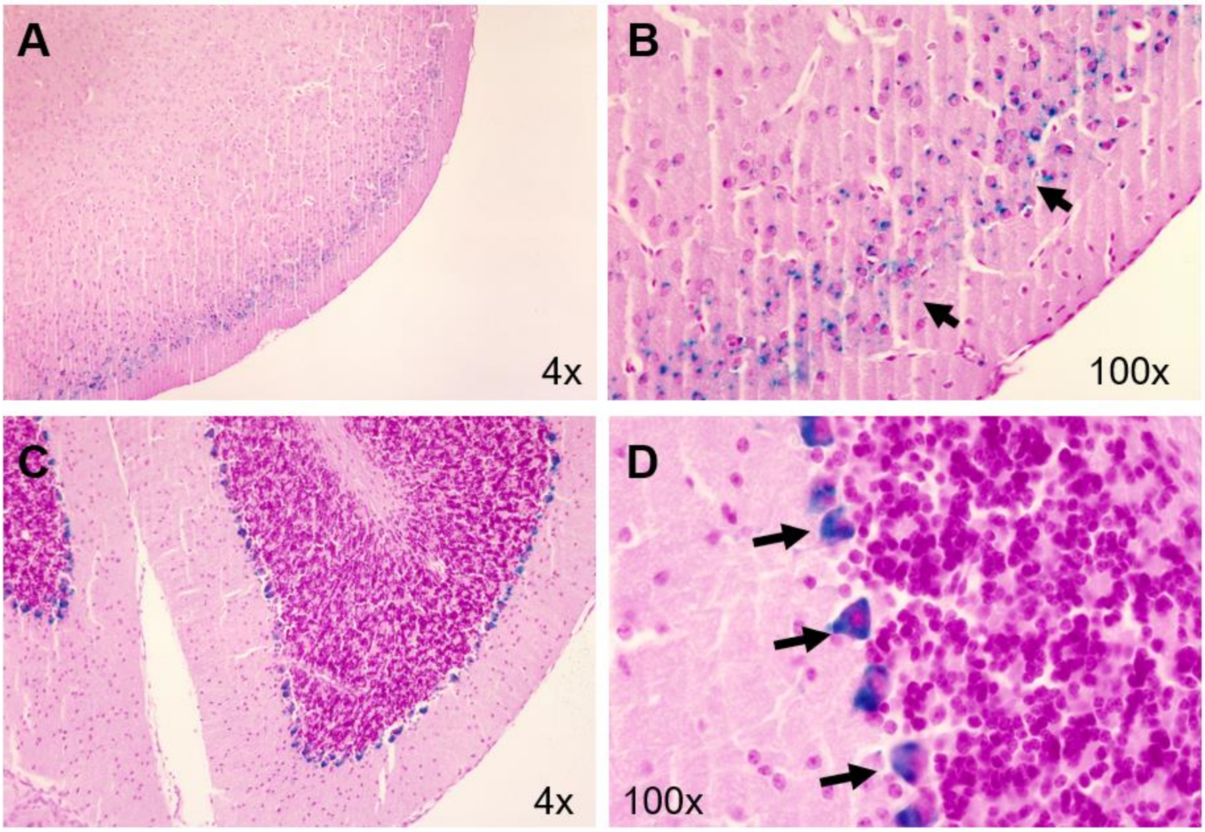

Figure S1. SOCS5 localisation in the brain. The SOCS5 coding region was replaced with the *lacZ* gene under the SOCS5 promoter as described previously [20]. Brain sections were fixed in 4% paraformaldehyde and stained for β-galactosidase activity as described in [31]. Sections were counterstained with nuclear fast red. Strong staining (blue) was observed in neurons (A-B) in the cortex a short distance from the outer meningeal margin. Positive staining of all Purkinje neurons in the cerebellum (C-D). Panels B and D are magnified sections of A and C, respectively.

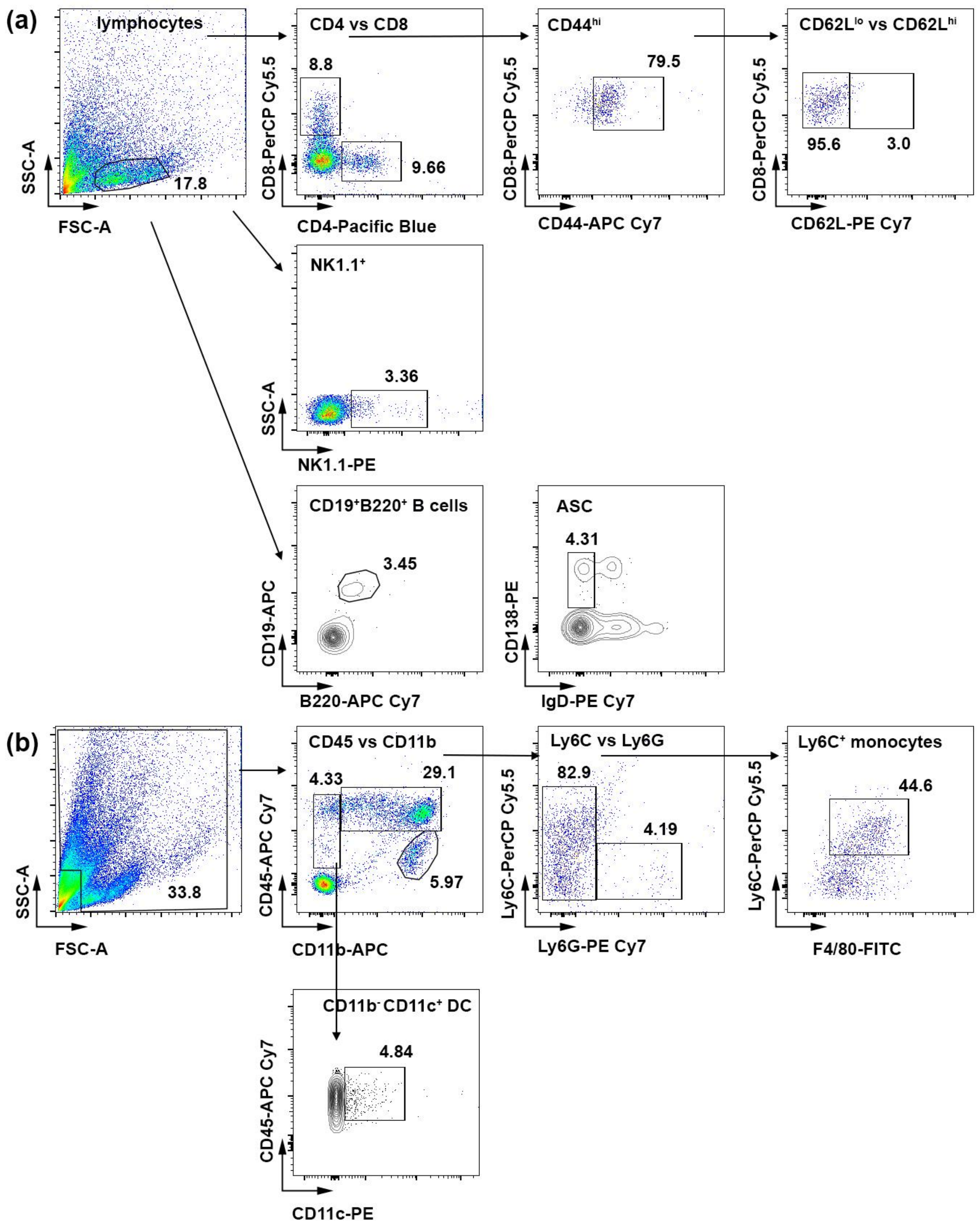

Figure S2. Gating strategy for flow cytometric analysis of brain infiltrates. Cells infiltrating the brain were purified on Percoll gradient as described in the Methods section. Combination of antibodies against surface markers (see Methods section) were used in separate panels to gate on a) T cells, NK cells and B cells, and b) innate cells. Values shown indicate the percentage of gated or positive cells. Representative FACS plots are shown.
